# Structure-function analysis of the EF-hand protein centrin-2 for its intracellular localization and nucleotide excision repair

**DOI:** 10.1093/nar/gkt434

**Published:** 2013-05-28

**Authors:** Ryotaro Nishi, Wataru Sakai, Daisuke Tone, Fumio Hanaoka, Kaoru Sugasawa

**Affiliations:** ^1^Biosignal Research Center, Organization of Advanced Science and Technology, Kobe University, Kobe, Hyogo 657-8501, Japan, ^2^Department of Biology, Graduate School of Science, Kobe University, Kobe, Hyogo 657-8501, Japan and ^3^Department of Life Science, Faculty of Science, Gakushuin University, Tokyo 171-8588, Japan

## Abstract

Centrin-2 is an evolutionarily conserved, calmodulin-related protein, which is involved in multiple cellular functions including centrosome regulation and nucleotide excision repair (NER) of DNA. Particularly to exert the latter function, complex formation with the XPC protein, the pivotal NER damage recognition factor, is crucial. Here, we show that the C-terminal half of centrin-2, containing two calcium-binding EF-hand motifs, is necessary and sufficient for both its localization to the centrosome and interaction with XPC. In *XPC*-deficient cells, nuclear localization of overexpressed centrin-2 largely depends on co-overexpression of XPC, and mutational analyses of the C-terminal domain suggest that XPC and the major binding partner in the centrosome share a common binding surface on the centrin-2 molecule. On the other hand, the N-terminal domain of centrin-2 also contains two EF-hand motifs but shows only low-binding affinity for calcium ions. Although the N-terminal domain is dispensable for enhancement of the DNA damage recognition activity of XPC, it contributes to augmenting rather weak physical interaction between XPC and XPA, another key factor involved in NER. These results suggest that centrin-2 may have evolved to bridge two protein factors, one with high affinity and the other with low affinity, thereby allowing delicate regulation of various biological processes.

## INTRODUCTION

Centrin, also known as caltractin, was initially identified in the flagellar apparatus of unicellular green alga (1–3). So far, centrin homologs have been identified in various species and are highly conserved from yeast to human. At least three centrin genes, *CETN1*, *CETN2* and *CETN3*, were identified in human (4–6), and all of these orthologs contain four EF-hand motifs, which are typical for calcium-binding proteins and share remarkable amino acid sequence homology with each other. Expression of centrin-1 seems to be restricted to testis, retina and differentiation stage of nasal epithelial cells where flagellated or ciliated cells exist, whereas centrin-2 and centrin-3 are expressed ubiquitously (7–11). These studies also revealed that centrin-2/3 localize to the centrosome in mammalian cells (12–15), and an essential role of centrin-2 in centriole duplication was reported from knockdown experiments in HeLa cells (14). Recently, several centrosomal proteins, such as POC5, Sfi1, CP110 and Galectin-3, have been reported to interact with centrin-2 in human cells, suggesting a central role of centrin-2 in centrosome regulation (16–19). Despite obvious localization to the centrosome, a majority of centrin-2 appears to exist in cytoplasm and nucleus, as suggested from cellular fractionation and immunofluorescent staining (13). In line with this knowledge, centrin-2 is associated with the nuclear pore through interaction with the Nup107-160 complex, which is involved in the nuclear export of mRNAs and proteins (20).

Nucleotide excision repair (NER) is a versatile DNA repair pathway, which can eliminate a wide variety of DNA lesions including ultraviolet light (UV)-induced photolesions as well as other bulky base adducts induced by various chemical compounds. Defects in NER are associated with several human autosomal recessive disorders, such as xeroderma pigmentosum (XP), Cockayne syndrome (CS) and trichothiodystrophy (TTD), which have been classified into several genetic complementation groups: eight for XP (XP-A through XP-G and XP-V), two for CS (CS-A and CS-B) and one for TTD (TTD-A). NER consists of two subpathways: one is global genome NER (GG-NER) operating throughout the genome, and the other is transcription-coupled NER that specifically removes transcription-blocking lesions. The XPC protein forms a heterotrimeric complex with one of the two mammalian homologs of *Saccharomyces cerevisiae* RAD23 (RAD23A/B) and centrin-2 (21–23), which plays an essential role in DNA damage recognition initiating GG-NER (24–26). This complex functions as a versatile damage recognition factor, as it senses the presence of oscillating unpaired normal bases in DNA duplex (27–29). Especially for UV-induced photolesions, on the other hand, another damage recognition factor DDB1/DDB2 (XPE), also known as the UV-damaged DNA-binding protein (UV-DDB), efficiently recognizes and binds to the damaged sites and vitally recruits XPC (30,31). The CUL4 ubiquitin ligase associated with UV-DDB (CRL4^DDB2^) is then activated to catalyze polyubiquitylation of XPC and DDB2, which we proposed is crucial for damage handover from UV-DDB to XPC (32,33). Regardless of the presence or absence of the intervention by UV-DDB, XPC recruits the TFIIH complex likely through their direct physical interaction (34–36). Two helicase subunits of TFIIH, XPB and XPD, locally unwind the DNA duplex around the damaged site presumably in concert with XPA and RPA (37). We have recently shown that the XPD helicase together with XPA scans along a DNA strand to verify the presence and location of altered DNA chemical structures (38). The open complex formation is crucial for the following dual incision by two NER endonucleases, ERCC1-XPF and XPG, and subsequent DNA repair synthesis.

Although centrin-2 was shown to be dispensable for reconstitution of the *in vitro* NER reaction, we have previously reported that centrin-2 enhances the damaged-DNA binding activity of XPC, resulting in acceleration of the NER reaction (39). However, precise roles for centrin-2, particularly relationships between its apparently diverse functions in GG-NER and the centrosome duplication, have remained to be understood. Here, we describe the structure-function relationship of centrin-2, which provides a novel insight into this multifunctional protein.

## MATERIALS AND METHODS

### Cell lines and culture

All human cell lines were cultured at 37°C in a humidified atmosphere containing 5% CO_2_. Simian virus 40-transformed human cell lines, normal (WI38 VA13) and XPC-deficient (XP4PASV), were cultured in Dulbecco’s modified Eagle’s medium supplemented with 10% fetal bovine serum. An insect cell line, High Five, was cultured at 27°C in Ex-Cell 405 medium (SAFC Biosciences).

Stable transformants of XP4PASV cells expressing FLAG-tagged XPC, wild-type (WT) or centrin-2-binding mutant (CBM), were established as described previously (39) and cultured in Dulbecco’s modified Eagle’s medium containing 10% fetal bovine serum and 10 µg/ml hygromycin B (Life Technologies).

### Preparation of cell extracts

For immunoprecipitation and immunoblot experiments, a monolayer of cells, typically grown in a 60-mm dish, were lysed on ice for 1 h with 500 µl of CSK buffer [10 mM Pipes (pH 6.8), 3 mM MgCl_2_, 1 mM EGTA, 0.1% Triton X-100, 10% glycerol, 0.25 mM phenylmethylsulfonyl fluoride and a protease inhibitor cocktail (Complete, EDTA-free: Roche Diagnostics)] containing 0.3 M NaCl. After the lysate was scraped into a 1.5-ml centrifuge tube, the culture dish was washed with 500 µl of the same buffer, which was combined with the recovered lysate. A soluble protein fraction was obtained by centrifugation at 20 000*g* for 15 min.

### Preparation of recombinant proteins

A heterodimeric complex containing FLAG-XPC and RAD23B-His was purified as described previously (39). To prepare the biotinylated XPC/RAD23B complex, the AviTag sequence was inserted between the FLAG-tag and XPC protein sequences. The resulting FLAG-AviTag-XPC protein was expressed by using the Bac-to-Bac baculovirus expression system (Life Technologies) and purified as a complex with RAD23B-His according to the standard procedures for the FLAG-XPC complex. The purified protein complex was biotinylated *in vitro* with biotin ligase (BirA enzyme; purchased from Avidity) as described in the Supplementary data. Other human NER proteins including TFIIH, FLAG-XPA, RPA, ERCC1-His/XPF and XPG were also purified as described previously (33,38).

For bacterial expression of glutathione *S*-transferase (GST)-tagged centrin-2, cDNA encoding WT or mutant centrin-2 was cloned into the pGEX-6P-1 vector (GE Healthcare Biosciences). An *Escherichia coli* strain BL21 (DE3) was used to express the GST-tagged centrin-2 proteins, which were then purified by using GSTrap FF and HiTrap Phenyl FF columns (GE Healthcare Biosciences). Detailed purification procedures are described in the Supplementary data. Where indicated, the GST-tag was removed by treatment with PreScission protease (GE Healthcare Biosciences).

### Pull-down assays

To assess the interaction of XPC with other NER factors, biotinylated XPC/RAD23B complex (0.2 µg) was first incubated on ice for 1 h with 20 µg of streptavidin-coated paramagnetic beads (Dynabeads M-280 Streptavidin: Life Technologies) in 20 µl of buffer A [20 mM sodium phosphate (pH 7.8), 1 mM EDTA, 10% glycerol, 0.1 mM dithiothreitol (DTT), 0.01% Triton X-100, 0.25 mM phenylmethylsulfonyl fluoride] containing 1.5 M NaCl, with or without centrin-2 in at least three times molar excess. After the unbound proteins were washed out, the beads were resuspended in 20 µl of buffer B [40 mM Hepes–NaOH (pH 7.9), 2 mM ATP, 3 mM MgCl_2_, 0.15 M NaCl, 5% glycerol, 0.5 mM DTT, 0.01% Triton X-100, 0.1 mg/ml bovine serum albumin] containing purified TFIIH (70 ng) and/or FLAG-XPA (130 ng) and incubated on ice for 1 h. The beads were then washed three times with 50 µl of the same buffer without ATP and heated at 95°C for 2 min in SDS sample buffer. To detect the interaction between XPA and centrin-2, FLAG-XPA (0.5 µg) was incubated on ice for 1 h with 100 µg of anti-DDDDK-tag Magnetic beads (Medical & Biological Laboratories) in 20 µl of buffer A containing 0.3 M NaCl. After the unbound proteins were washed out, the beads were resuspended in 20 µl of buffer B containing centrin-2 and incubated on ice for 1 h. The beads were then washed three times with 50 µl of buffer B minus ATP, and the bound proteins were eluted by incubation on ice for 1 h with 20 µl of the same buffer containing 500 µg/ml of FLAG peptide (Sigma-Aldrich).

### Immunoprecipitation from cell extracts

For transient overexpression of FLAG-XPC or HA-centrin-2 in human cells, cDNA encoding the corresponding protein was cloned into the pCAGGS vector (40). The constructs for FLAG-XPC and HA-centrin-2 (WT or mutant) were transfected into XP4PASV cells in 60-mm culture dishes by using FuGENE6 (Roche Diagnostics). Because of considerable difference in relative protein expression levels, the amounts of DNA used for transfection were varied: 2 µg (FLAG-XPC), 1 µg (HA-centrin-2, WT and point mutants M1-M4), 3 µg (HA-centrin-2, N), and 4 µg (HA-centrin-2, C), respectively. At 36 h post-transfection, soluble cell extracts were prepared as described earlier in the text. Each extract containing 100 µg of total proteins was mixed with 25 µl of either anti-FLAG M2 (Sigma-Aldrich) or anti-HA (3F10: Roche Diagnostics) agarose beads and rotated at 4°C overnight. The beads were then successively washed twice with CSK buffer containing 0.3 M NaCl, three times with CSK buffer containing 1 M NaCl, and once with CSK buffer containing 0.3 M NaCl. Bound proteins were eluted by incubating on ice for 30 min with 50 µl of CSK buffer containing 0.3 M NaCl and 500 µg/ml of FLAG peptide (for FLAG) or by heating at 95°C for 5 min in SDS sample buffer (for HA).

### Biochemical assays

Electrophoretic mobility shift assays (EMSA) were carried out by using the ^32^P-labeled, 180 bp DNA fragment with or without a site-specific single 6-4PP as described previously (39). For *in vitro* NER dual incision assays, the internally ^32^P-labeled cccDNA substrate containing a site-specific 6-4PP was prepared as described previously (28). The standard reaction mixture (15 µl) contained 40 mM Hepes–KOH (pH 7.8), 70 mM NaCl, 0.5 mM DTT, 5 mM MgCl_2_, 0.5 mM EDTA, 2 mM ATP, bovine serum albumin (9 µg), FLAG-XPA (270 ng), ERCC1-His/XPF (12.5 ng), RPA (100 ng), XPG (11.6 ng), TFIIH (290 ng), FLAG-XPC/RAD23B-His (with or without centrin-2) and the ^32^P-labeled DNA substrate (10^5 ^cpm). The reactions were pre-incubated at 30°C for 10 min without DNA and, on addition of the substrate, further incubated at 30°C for 1 h. DNA was purified from the reaction mixture and subjected to 10% denaturing PAGE followed by autoradiography.

### Chromatin immunoprecipitation

Typically, ∼1 × 10^7^ cells in a 150-mm dish were irradiated with UVC (10 J/m^2^) and cultured for various time. The cells were cross-linked at room temperature for 5 min with 1% formaldehyde. Excess formaldehyde was quenched by addition of glycine to a final concentration of 0.125 M. Following additional 5-min incubation, the cells were washed twice with cold PBS, collected and incubated in CSK buffer containing 0.3 M NaCl on a rotator for 20 min at 4°C. After centrifugation (400× *g*, 10 min, 4°C), the cell pellets were washed three times with the same buffer and subsequently resuspended in buffer C [10 mM Tris–HCl (pH 8.0), 140 mM NaCl, 0.1% SDS, 1% Triton X-100, 0.1% sodium deoxycholate, a protease inhibitor cocktail]. The suspension was sonicated on ice (16 cycles of 30 s on/30 s off, at 300 W) using Bioruptor (UCD-300, Cosmo Bio). After centrifugation (20 000× *g*, 20 min, twice), the resulting supernatant was used for chromatin immunoprecipitation (ChIP) as the cross-linked chromatin-enriched fraction. For each ChIP reaction, an equal amount of protein was immunoprecipitated using anti-FLAG M2 magnetic beads in buffer C at 4°C overnight. The beads were washed five times with 10 times volume of buffer C, and the bound materials were eluted with the FLAG peptide (500 μg/ml in buffer C) for 60 min on ice. Before SDS–PAGE and immunoblot analyses, the samples were de-cross-linked by incubation at 95°C for 45 min in SDS sample buffer.

### Antibodies

Anti-XPC (38,41), anti-centrin-2 (39) and anti-cyclobutane pyrimidine dimers (CPD) (TDM2) (42) antibodies were obtained as previously described. Anti-HA (12CA5) antibody was purified from ascites obtained from Balb/c mice transplanted with the antibody-producing hybridoma cell line. Anti-centrin (C-terminus: C7736, for immunoblotting of recombinant centrin-2), anti-α-tubulin (T6074), and anti-γ-tubulin (T6557) antibodies were purchased from Sigma-Aldrich. Anti-XPA (FL-273: sc-853) and anti-XPB (S-19: sc-293) antibodies were purchased from Santa Cruz Biotechnology. Anti-HA (3F10) (Roche Diagnostics), anti-GST (GE Healthcare Biosciences), anti-XPD (Abcam: ab54676) and anti-cyclin H (Cell Signaling: 2927) antibodies were also purchased, respectively. Alexa Fluor-labeled secondary antibodies (Alexa Fluor 405 for anti-rabbit IgG, Alexa Fluor 488 for anti-mouse IgG, Alexa Fluor 594 for anti-rat IgG) were purchased from Life Technologies.

### Other methods

Detailed procedures for immunofluorescent staining are described in the Supplementary data. Detection of immunoblotting was based on chemiluminescence using an appropriate secondary antibody conjugated to alkaline phosphatase (Sigma-Aldrich) or horseradish peroxidase (R&D Systems) in combination with the proper substrate reagent, CDP-Star (for alkaline phosphatase: Roche Diagnostics) or Immobilon Western (for horseradish peroxidase: Millipore). Images were taken with the ImageQuant LAS 4000 system (GE Healthcare Biosciences) and also with exposure of X-ray films (Fujifilm RX-U). Quantification was made with ImageQuant TL (GE Healthcare Biosciences) and/or ImageJ softwares.

## RESULTS

### The C-terminal half of centrin-2 is sufficient for interaction with XPC

**Figure 1.**
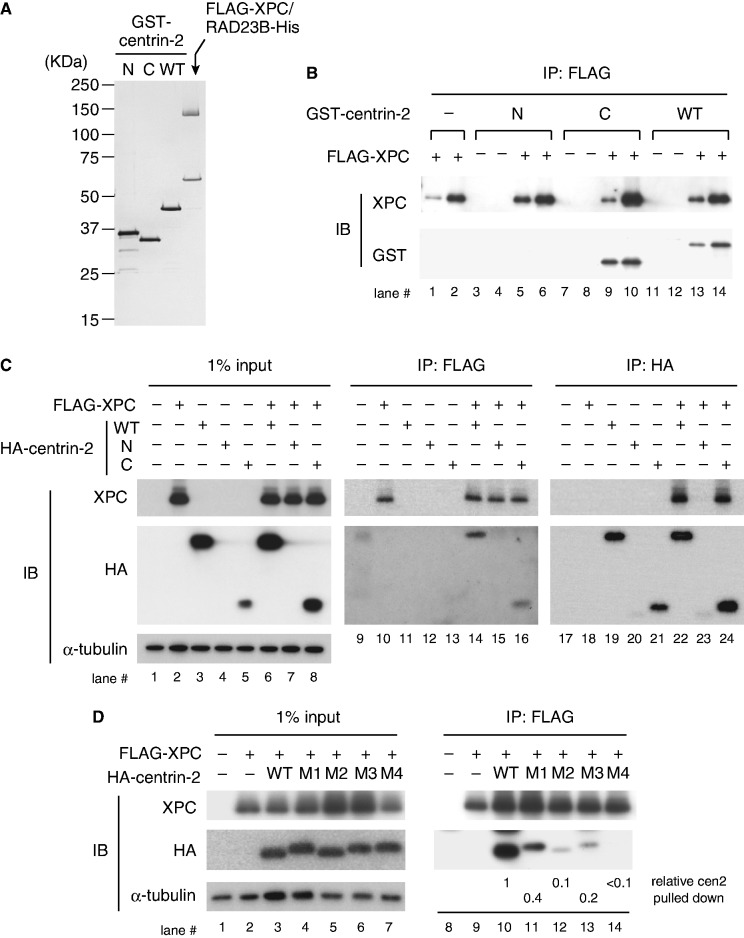
The C-terminal half of centrin-2 is necessary and sufficient for interaction with XPC. (**A**) Purified GST-centrin-2 (WT, N or C) and the FLAG-XPC/RAD23B-His complex (0.1 µg each) were subjected to SDS–PAGE followed by silver staining. (**B**) Purified GST-centrin-2 (WT, N or C) or GST alone (as a negative control) was incubated with FLAG-XPC/RAD23B-His and then pulled down with anti-FLAG antibody beads. Two different amounts (4 and 8%) of the precipitated proteins were subjected to immunoblotting with the indicated antibodies. (**C**) FLAG-XPC and HA-centrin-2 were transiently expressed in XP4PASV cells and solubilized proteins were subjected to immunoprecipitation with anti-FLAG and anti-HA (3F10) antibodies as indicated. The precipitated proteins, along with 1% of the input extracts, were subjected to immunoblotting with anti-XPC and anti-HA (12CA5) antibodies, respectively. (**D**) FLAG-XPC and HA-centrin-2 point mutants (M1–M4) were transiently expressed in XP4PASV cells and subjected to immunoprecipitation with anti-FLAG antibody. The amino acids changed to alanines in each mutant are as follows: M1, F113A/L133A; M2, F113A/M145A; M3, L112A/F113A/L133A; M4. L112A/F113A/L133A/M145A. For lanes 10–14, the amounts of centrin-2 pulled down were quantified, normalized with the amounts of XPC and expressed as relative values (the value of WT centrin-2 was set as 1).

Human centrin-2 can be divided into two domains, the amino-terminal (N) domain (amino acid residues 1-93) and the carboxyl-terminal (C) domain (amino acid residues 94–172), which contain each two EF-hand motifs and share remarkable sequence homology with each other. To test interaction with the XPC/RAD23B complex *in vitro*, WT and the individual domains of centrin-2 (N and C) were expressed and purified as GST-fusion proteins (Figure 1A). In line with the previous study using a synthetic peptide corresponding to the centrin-2-binding domain of XPC (43–45), our pull-down assays indicate that the C domain of centrin-2, as well as the WT protein, interacts with XPC, whereas no interaction was detected with the N domain (Figure 1B).

To further examine the interactions *in vivo*, FLAG-XPC and HA-tagged centrin-2 (WT, N or C) were ectopically co-expressed in XP4PASV cells, which lack expression of endogenous XPC. Immunoprecipitation with anti-FLAG and anti-HA antibodies revealed that the C domain of centrin-2 is sufficient for interaction with XPC (Figure 1C). However, expression of the N domain was poor, presumably owing to *in vivo* instability of the truncated protein (lanes 4 and 7). Based on the structural study by others (43), we predicted four amino acid residues in the C domain of centrin-2 (L112, F113, L133 and M145) that may be involved in the interaction with XPC, and generated several mutant centrin-2 (designated as M1 through M4), in which the four amino acids were replaced by alanines in various combinations. As expected, these mutant proteins were expressed as well as the WT centrin-2 and showed dramatically reduced binding to XPC (Figure 1D). Especially, no interaction could be detected with the M4 mutant carrying quadruple alanine substitution, despite the presence of the intact N domain. These results indicate that the C domain of centrin-2 is sufficient for interaction with full-length XPC, whereas contribution of the N domain is negligible.

### Nuclear localization and recruitment to UV lesions of centrin-2 depend on interaction with XPC

**Figure 2.**
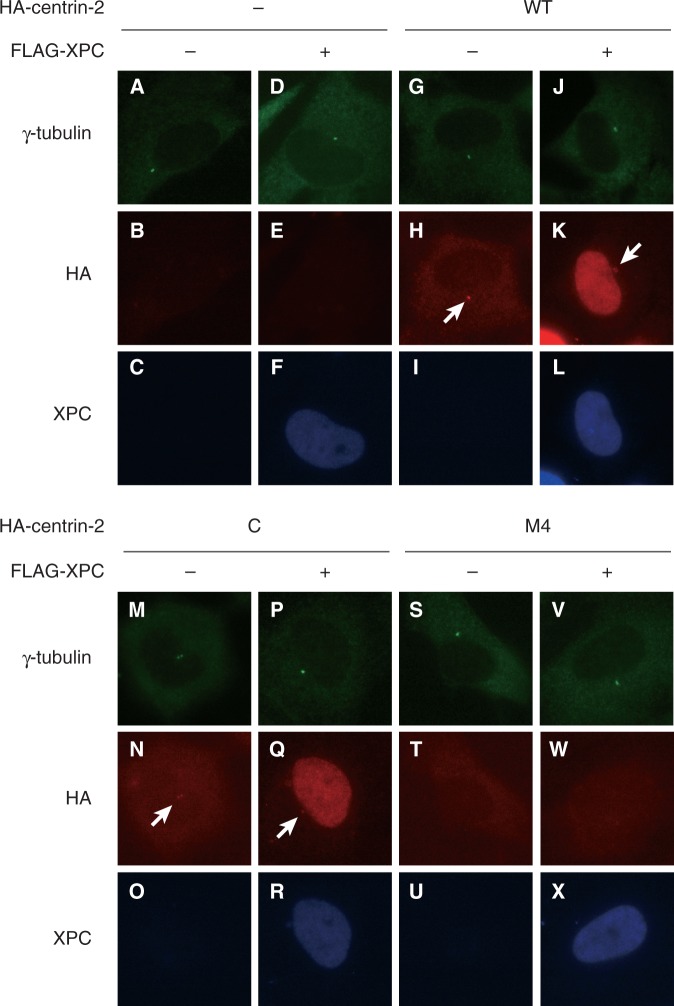
Nuclear and centrosomal localization of the WT and mutant centrin-2 proteins. FLAG-XPC and/or HA-centrin-2 (WT, C or M4) were transiently expressed in XP4PASV cells in various combinations. The transfected cells were subjected to triple immunofluorescent staining with anti-γ-tubulin (green), anti-HA (3F10) (red) and anti-XPC (blue) antibodies as indicated. The arrows indicate centrosomal localization of HA-centrin-2.

It has been reported that endogenously-expressed centrin-2 localizes not only in the centrosome but also in cytoplasm and nucleus (15). By using immunofluorescent staining, we examined subcellular distribution of the ectopically expressed HA-centrin-2 (WT or mutant) in the presence or absence of co-expression of FLAG-XPC (Figure 2). In the absence of XPC, WT centrin-2 clearly localized in the centrosome, whereas dispersed signals were also visible throughout the cell (Figure 2H). When co-expressed with XPC, however, nuclear distribution of centrin-2 became dramatically pronounced with the remaining presence in the centrosome (Figure 2K). Similar distribution patterns were observed with the C domain of centrin-2 (Figure 2N and Q), whereas the M4 mutant localized neither in the centrosome nor in nucleus even in the presence of XPC (Figure 2W). Thus, centrin-2 probably uses a common interface for interactions with XPC and the centrosome, although its binding partner in the centrosome is different from XPC.

**Figure 3.**
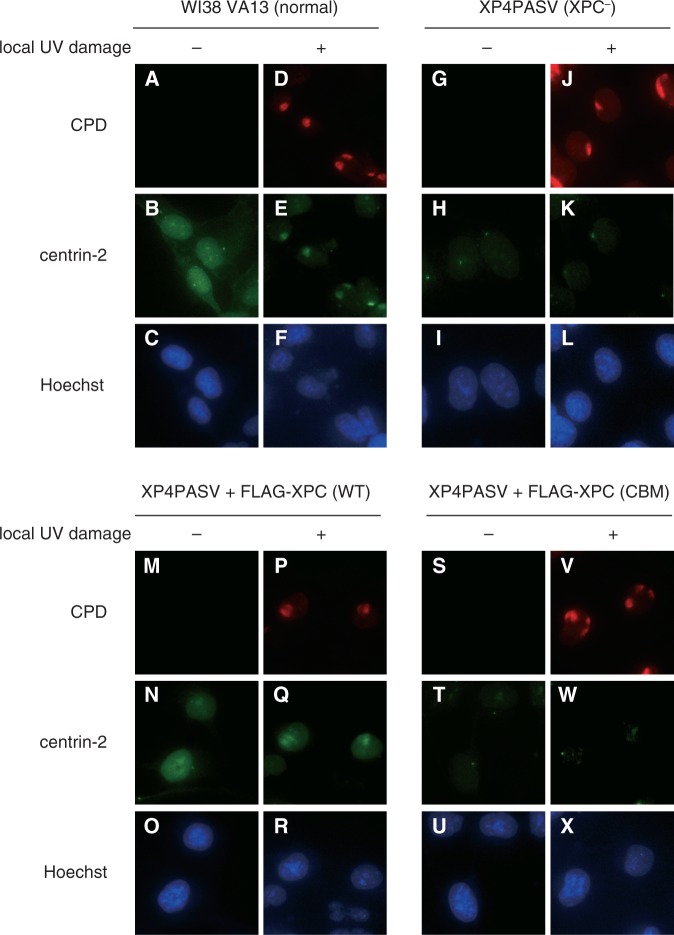
Subcellular localization of endogenously-expressed centrin-2. Indicated cell lines were subjected to double immunofluorescent staining with anti-CPD (red) and anti-centrin-2 (green) antibodies with or without localized UVC irradiation. The nuclear DNA was counter-stained with Hoechst 33342.

Centrosomal localization of the ectopically expressed centrin-2 was not significantly altered by global UVC irradiation of the cells, regardless of the presence or absence of XPC (Supplementary Figure S1: compare with Figure 2, panels A through L). We further investigated behaviors of the mutant centrin-2 on local UVC irradiation through micropore membrane filters. As shown in Supplementary Figure S2, the C domain of centrin-2, but not the M4 mutant, co-localized with CPD as well as co-expressed XPC, exactly as observed with WT centrin-2. Notably, immunofluorescent analyses of endogenously expressed centrin-2 further verified the aforementioned results (Figure 3). Nuclear localization and recruitment to local UV lesions of endogenous centrin-2 could be observed in normal human fibroblasts WI38 VA13, but not in XP4PASV cells. These phenotypes were corrected by stable expression of WT XPC, but not of XPC (CBM), the mutant XPC containing three amino acid substitutions that compromise interaction with centrin-2 (39). On the other hand, centrosomal localization of centrin-2 was still detectable in the absence of XPC. Taken together, these results indicate that nuclear localization of centrin-2 largely depends on interaction with XPC through its C domain, and that the C domain alone can be recruited to DNA damage sites together with XPC.

### The C domain of centrin-2 is sufficient to enhance damaged DNA binding by XPC and *in vitro* NER dual incision

**Figure 4.**
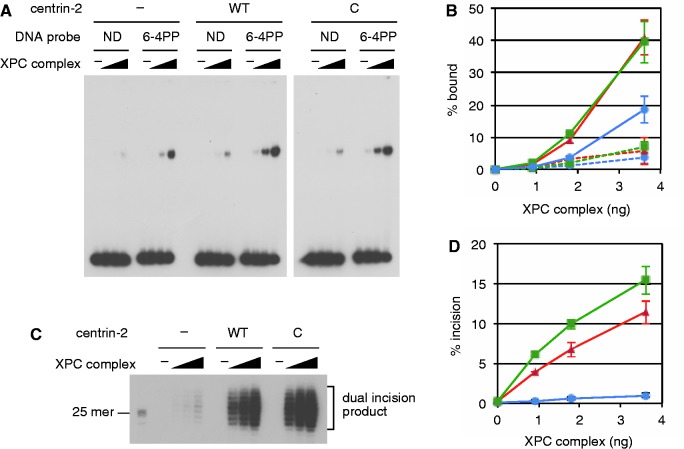
The C domain of centrin-2 is sufficient to enhance both damaged DNA binding and NER dual incision activities of XPC. (**A**) EMSA was carried out, in which the DNA substrate (0.35 nM) with or without a single site-specific 6-4PP was included. Three different concentrations of XPC/RAD23B, with or without centrin-2 (WT or C), were used. (**B**) Percentage of the shifted DNA probe was calculated from each lane in panel A and plotted as a graph. Solid and broken lines represent the 6-4PP-containing and non-damaged (ND) DNA probes, respectively. Blue: no centrin-2, red: WT centrin-2, green: the C domain of centrin-2. (**C**) *In vitro* NER dual incision assays were carried out with purified NER proteins and internally ^32^P-labeled DNA substrate including a single 6-4 PP. Only part of the autoradiography containing dual incision products is shown. Exactly the same amounts of the XPC complex as used in panel A were included in the reactions. (**D**) Percentage of the incised DNA substrate was calculated from each lane in panel (C) and plotted as a graph. Blue: no centrin-2, red: WT centrin-2, green: the C domain of centrin-2. For panels (B) and (D), the mean values and standard errors were calculated from two independent experiments, respectively.

Roles for the centrin-2 domains in NER were further studied with biochemical assays. First, DNA-binding activity of XPC was assessed by EMSAs using a ^32^P-labeled double-stranded DNA fragment with or without a site-specific (6-4) photoproduct (6-4PP). To allow accurate comparison, aliquots of the purified FLAG-XPC/RAD23B-His protein immobilized on anti-FLAG magnetic beads were incubated in the presence or absence of centrin-2 (WT or C domain, detached from the GST tag). After extensive washing, the bound protein complexes were eluted by incubating with the FLAG peptide. Based on immunoblot analyses, we confirmed that the two reconstituted heterotrimers (with either WT or C domain of centrin-2) as well as the control heterodimer contained almost the same concentrations of XPC (Supplementary Figure S3). In line with our previous findings (39), the complex formation with WT centrin-2 significantly enhanced damage-specific DNA binding of XPC (Figure 4A). Notably, the C domain alone of centrin-2 was sufficient to augment the damaged DNA-binding activity of XPC to a comparable extent (see quantitative data in Figure 4B). Although the non-tagged N domain could not be tested because of its instability, another experiment with GST fusion proteins indicated that the DNA binding of XPC was unaffected by the presence of the N domain, as expected (Supplementary Figure S4).

We further investigated how the centrin-2 domains affect *in vitro* NER reactions (Figure 4C). To avoid interference by centrin-2 present in crude cell extracts, NER dual incision was reconstituted with purified NER factors, XPC/RAD23B, TFIIH, XPA, RPA, ERCC1/XPF and XPG, all from recombinant sources. Although centrin-2 itself is known to be dispensable for *in vitro* NER, excision of the oligonucleotides containing a 6-4PP was dramatically increased by the presence of WT centrin-2, as described previously (39). Compared with the somewhat weak (∼2-fold) enhancement of the XPC binding to 6-4PPs, the stimulation of dual incision was much more pronounced (>10-fold; compare Figure 4B and D). Notably, the C domain was also capable of stimulating the dual incision even to a slightly higher extent than the WT centrin-2. These results indicate that centrin-2 not only augments the damage recognition activity of XPC but also affects the repair process following the DNA binding by XPC.

### Centrin-2 augments the interaction between XPC and XPA

**Figure 5.**
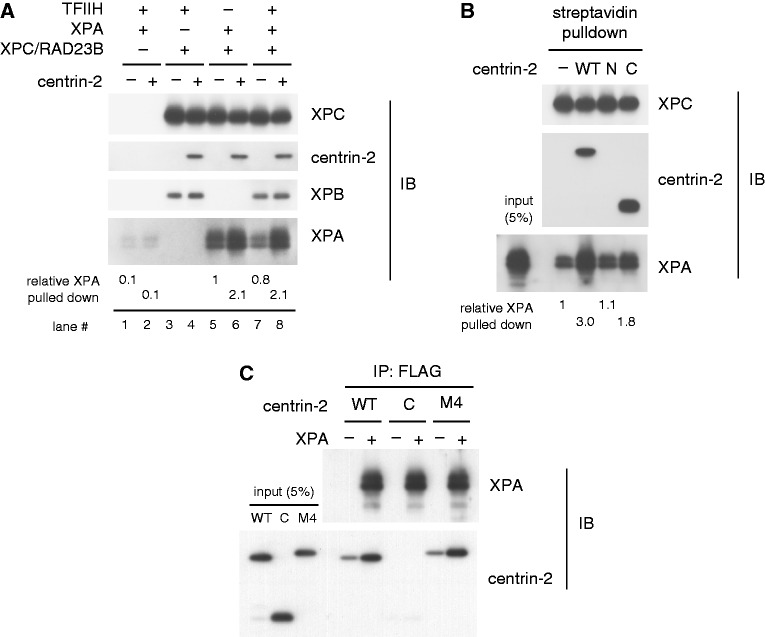
Centrin-2 augments physical interaction between XPC and XPA. (**A**) Biotinylated XPC/RAD23B immobilized on paramagnetic beads was used to pull down purified TFIIH and/or XPA in the presence or absence of WT centrin-2. (**B**) XPA was pulled down with paramagnetic beads pre-bound to XPC/RAD23B with or without centrin-2 (WT, N or C). The amounts of XPA pulled down were quantified and shown as relative values (average values were calculated from two experiments). (**C**) Centrin-2, WT, C domain or M4 mutant, was pulled down with FLAG-XPA immobilized on anti-DDDDK-tag Magnetic beads. In each experiment, co-precipitated proteins were subjected to immunoblotting with the indicated antibodies.

Based on the aforementioned results, we next examined possible effects of centrin-2 on protein–protein interactions. For this purpose, biotinylated XPC/RAD23B was immobilized on streptavidin-coated paramagnetic beads and incubated in the presence or absence of WT centrin-2. After a wash, the beads bound to XPC (either heterodimer or heterotrimer) were then used to pull down purified recombinant TFIIH and/or XPA (Figure 5A). Strong physical interactions have been reported between XPC and TFIIH, for which the XPB and p62 subunits in TFIIH are likely responsible (34–36). In our experimental conditions, TFIIH bound to XPC in a nearly quantitative manner so that little difference was observed in the amount of bound XPB, regardless of the presence or absence of centrin-2 (Figure 5A, lanes 3 and 4; see also Supplementary Figure S5). Furthermore, in line with a previous report (46), XPA also showed direct interaction with XPC/RAD23B. Although this interaction seemed much weaker than that of TFIIH, it was significantly enhanced by the presence of centrin-2 (lanes 5 and 6). Essentially, the same results were obtained when TFIIH and XPA were added simultaneously (lanes 7 and 8). Intriguingly, such an effect on the XPC–XPA interaction was partially compromised, if the C domain of centrin-2 was used instead of the full-length protein (Figure 5B). We further demonstrated the direct interaction of XPA with WT centrin-2, but not with the C domain alone, in the absence of XPC/RAD23B. Although the N domain alone could not be tested in this experiment because of its instability without the GST tag, the M4 mutant of centrin-2 retaining the intact N domain also interacted with XPA (Figure 5C). Both the augmentation of the XPC–XPA interaction and the direct interaction with XPA were compromised if centrin-2 retained the GST tag on its N-terminus, also pointing to involvement of the N-terminal part of centrin-2 (data not shown).

**Figure 6.**
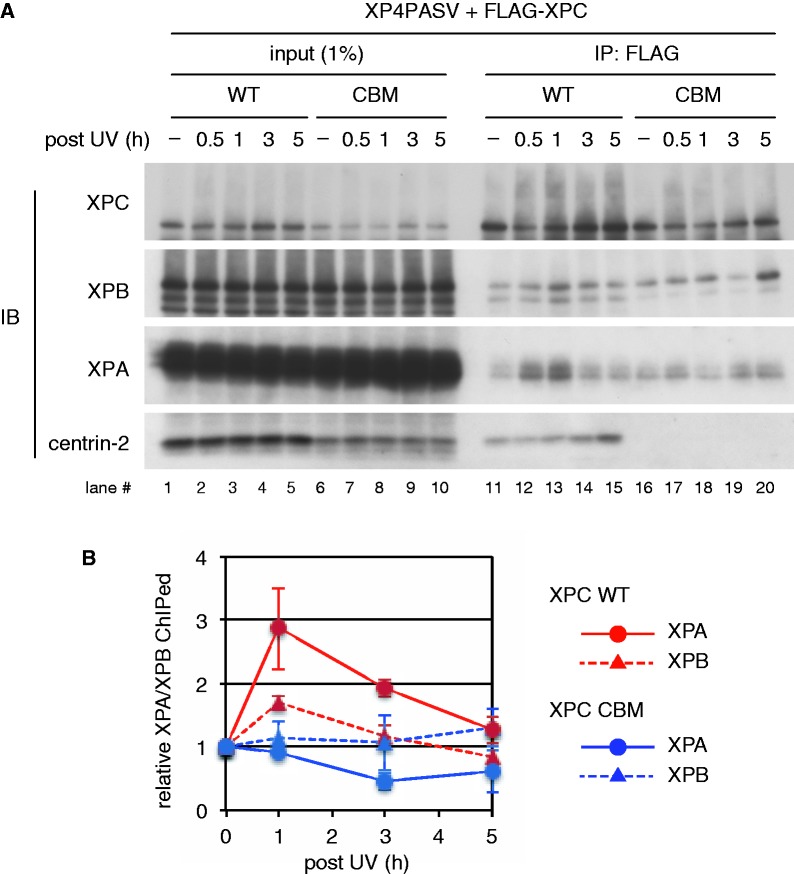
Centrin-2 is required for efficient UV-induced recruitment of XPA to chromatin sites where XPC is bound. Two stably transformed cell lines of XP4PASV expressing FLAG-XPC (WT or CBM) were treated with UVC (10 J/m^2^). (**A**) Cross-linked chromatin fragments were prepared at various time points and subjected to ChIP assays with the anti-FLAG M2 magnetic beads. Co-precipitated proteins were detected by immunoblot analyses using the indicated antibodies. (**B**) The amounts of XPB and XPA in the ChIP fractions were quantified and plotted as relative values (the values from non-irradiated samples were set as 1). Average values and standard errors were calculated from three independent experiments.

The aforementioned results suggest that centrin-2 bound to XPC may contribute to promoting the assembly of NER factors in relatively early stages of the repair process. To test this possibility *in vivo*, we carried out ChIP experiments with the stably transformed cell lines of XP4PASV, which expressed FLAG-XPC (either WT or CBM) at physiological levels. We have previously described that the cell line expressing XPC (CBM) showed significantly slower repair of UV-induced 6-4PPs than the cells expressing WT XPC (39). After each cell line was exposed to UVC, formaldehyde-fixed chromatin was prepared at various time points, fragmented by sonication, and subjected to immunoprecipitation with the anti-FLAG antibody. As shown in Figure 6A, both the WT and CBM mutant of XPC were cross-linked to chromatin in a nearly constitutive manner, even without the UV irradiation (see lanes 1 through 10, for input chromatin fractions). Although the two XPC proteins were expressed at comparable levels and solubilized with salts in a similar manner (Supplementary Figure S6), the amounts of cross-linked XPC (CBM) were reproducibly lower than the WT protein. In the ChIP samples targeting FLAG-XPC, XPB was detected constitutively, regardless of the types of XPC expressed, likely reflecting the strong physical interaction between XPC and TFIIH. Although the co-precipitation of XPB appeared to increase only slightly after exposure to UV, robust and transient association with XPA was induced with WT XPC, peaking ∼30–60 min after UV (lanes 12 and 13). In a marked contrast, the UV-induced assembly of XPA into the complex with XPC (CBM) seemed to be attenuated substantially (lanes 16 through 20: see also the quantitative data in Figure 6B). These results support the notion that one of the roles for centrin-2 may be promoting recruitment of XPA to the XPC-TFIIH complex pre-assembled at damaged DNA sites.

## DISCUSSION

### The C domain of centrin-2 is necessary and sufficient for both XPC binding and centrosomal localization

Previous structural and thermodynamical studies showed that only the C-terminal half of centrin-2 interacts with a peptide corresponding to the centrin-2-binding site in XPC, whereas the remaining N domain seemed to be devoid of any interaction (43–45). In the present study, we biochemically confirmed that the C domain of centrin-2 is exclusively responsible for the interaction with the intact XPC protein and identified four hydrophobic amino acid residues (L112, F113, L133 and M145) that are critical for the interaction (Figure 1D). Notably, changes of the four amino acids in the C domain compromised not only binding to XPC but also *in vivo* localization to the centrosome, even though the intact N domain is present (Figure 2). Because centrin-2 can localize to the centrosome in the XPC-deficient cells as well, major binding partners that anchor centrin-2 to the centrosome must be different from XPC. So far several interacting proteins have been reported for centrin-2, some of which have been shown to localize in the centrosome (such as hPOC5, hSfi1 and Galectin-3) (16–19). Our results strongly suggest that XPC shares the same binding surface with such centrosomal targets of centrin-2.

Apart from the centrosomal localization, co-expression of XPC dramatically altered intracellular distribution of centrin-2. In the absence of XPC, centrin-2 (both endogenously and ectopically expressed) appears to disperse throughout the cell, whereas expression of XPC results in remarkable accumulation of centrin-2 in the nucleus. This does not necessarily mean that, under physiological conditions, the nuclear localization of centrin-2 solely depends on XPC: other target proteins may be present within the nucleus, which could be expressed much less abundantly than XPC or have lower binding affinity for centrin-2. Indeed, centrin-2 has been suggested to be involved in other nuclear functions, such as nuclear membrane transport (20) and homologous recombination (47). Nevertheless, the subcellular distribution of centrin-2 was not substantially affected by global UV irradiation, regardless of the presence or absence of XPC (compare Figure 2 with Supplementary Figure S1), arguing against the possibility that DNA damage may induce drastic translocation of centrin-2 (e.g. from cytoplasm and/or the centrosome into nucleus). Taken together, although dynamic association/dissociation modes (i.e. shuttling between the nucleus and the centrosome) of centrin-2 are not excluded, our results indicate that the centrosomal functions of centrin-2 are largely independent of NER.

### Roles for the centrin-2 domains in the NER damage recognition mechanism

We have previously shown that complex formation with centrin-2 enhanced both affinity and specificity of the damaged DNA-binding by XPC/RAD23B (39). In the present study, we show that the C domain of centrin-2 is exclusively responsible for enhancing the XPC damage recognition activity, whereas the N domain is dispensable. So far, no DNA-binding activity associated with centrin-2 itself has been reported (see also Supplementary Figure S4). The X-ray crystal structure of the *S.cerevisiae* XPC homolog RAD4 revealed several functional domains, the transglutaminase-homology domain (TGD) and three β-hairpin domains (BHD1, 2, 3), which are crucial for its damage recognition activity (27). Although the α-helix in XPC targeting centrin-2 does not directly overlap with any of these corresponding domains, it is possible that the interaction with the C domain of centrin-2 may have a rather widespread impact on the conformation of XPC, thereby allowing better recognition of DNA sites containing unpaired bases. With a C-terminal fragment of human XPC (amino acids 815-940), only a minor conformational change upon binding to centrin-2 was detected by NMR (48). However, it should be noted that this truncated XPC did not involve all the necessary domains (transglutaminase-homology domain and β-hairpin domains) for damaged DNA binding. In this regard, it must be relevant if a yeast counterpart of centrin-2 could be added to the solved structure of the DNA-RAD4 complex. In budding yeast, CDC31 has been known as a centrin homolog, whereas another homolog RAD33 has recently been implicated in NER, which binds RAD4 through similar hydrophobic interactions (49,50). Although structural impacts of RAD33 on the DNA-RAD4 complex would shed light on this issue, the structure of XPC still deserves to be solved, considering functional difference between XPC (involved only in GG-NER) and RAD4 (both GG-NER and transcription-coupled NER).

Centrin-2 also stimulated the NER dual incision reaction in the highly defined system reconstituted with recombinant protein factors. Although the enhancement of the XPC damage recognition activity likely made a certain contribution, the observed stimulation of dual incision was beyond the levels expected from the DNA binding assays, suggesting additional functions of centrin-2 after DNA binding. Here, we report intervention of centrin-2 in the XPC–XPA interaction, proposing as a candidate for such novel functions. There has been accumulating evidence that, in the NER process, the presence and location of a proper DNA lesion are recognized and verified through multiple steps (28,38,51,52). To induce a productive repair reaction, XPC first recognizes and interacts with unpaired normal bases in the undamaged DNA strand. This allows TFIIH, which is recruited likely through the interaction with XPC, to be loaded, such that the XPD helicase subunit binds to the opposite damaged strand. Then the XPD helicase moves along the DNA strand in 5′–3′ direction, and dual incision occurs only when this translocation is hampered by aberrant chemical structures of DNA (38,53,54). Notably, our previous biochemical studies indicated that XPA was required for triggering the damage search by XPD, suggesting assembly and translocation of the ternary complex containing XPC, TFIIH and XPA (38). Although XPA has been supposed to be recruited to DNA damage mainly through the reported interaction with TFIIH (55,56), centrin-2 may contribute to the XPA recruitment and/or stabilization of the ternary complex.

Our data strongly suggest that the N domain of centrin-2 is mainly involved in the interaction with XPA. However, in the defined dual incision system, the C domain alone showed a comparable (or even slightly higher) activity than WT centrin-2. Although *in vivo* recruitment of XPA to XPC-bound chromatin sites appeared to be compromised in the absence of centrin-2, the C domain of centrin-2 may have still unknown functions, which stimulate the NER reaction more prominently. In addition, the N domain may have both positive and negative effects on NER at least *in vitro*, considering its reported self-assembling properties (57). Despite the remarkable homology of the amino acid sequences, it was reported that the two domains of centrin-2 form different structures. The EF-hand motifs in the C domain adopt a typical structure for the calmodulin super family that has a hydrophobic pocket and shows high-affinity binding to calcium ions, whereas the N domain forms a closed structure with only low affinities for calcium ions (43,44,57). Although calmodulin typically holds an α-helix in target proteins with both the N- and C-terminal EF-hand domains (58–60), it binds certain targets only through its C-terminal domain (61), and our results suggest that centrin-2 may have evolved to be more specialized to bridge between two proteins, one with high affinity and the other with low affinity. It is possible that such low affinity (and probably transient) interaction may allow fine regulation of biological processes, not only in NER but also in the centrosomes and other cellular functions. Further studies would shed light on the precise roles for this multifunctional protein.

## SUPPLEMENTARY DATA

Supplementary Data are available at NAR Online: Supplementary Figures 1–6 and Supplementary Methods.

## FUNDING

Ministry of Education, Culture, Sports, Science and Technology of Japan (to R.N., F.H. and K.S.) and the Solution Oriented Research for Science and Technology (SORST) from the Japan Science and Technology Agency (to F.H. and K.S.). Funding for open access charge: Grant-in-Aid for Scientific Research from the Ministry of Education, Culture, Sports, Science and Technology of Japan (to K.S.).

*Conflict of interest statement.* None declared.

## Supplementary Material

Supplementary Data
