# Supplementary material for: Structure-function analysis of the EF-hand protein centrin-2 for its intracellular localization and nucleotide excision repair
Source: Nucleic Acids Res. 2013 May 28;41(14):6917–29. doi: 10.1093/nar/gkt434 (PMC3737541; doi:10.1093/nar/gkt434)
Supplement: Supplementary Data [file supp_41_14_6917__index.html]

Structure-function analysis of the EF-hand protein centrin-2 for its intracellular localization and nucleotide excision repair — Supplementary Data 

# Structure-function analysis of the EF-hand protein centrin-2 for its intracellular localization and nucleotide excision repair

## Supplementary Data

files

**Files in this Data Supplement:**

- Supplementary Data - pdf file
