# Supplementary material for: Structure-function analysis of the EF-hand protein centrin-2 for its intracellular localization and nucleotide excision repair
Source: Nucleic Acids Res. 2013 May 28;41(14):6917–29. doi: 10.1093/nar/gkt434 (PMC3737541; doi:10.1093/nar/gkt434)
Supplement: Supplementary Data [file supp_gkt434_Supplemental_data_3.pdf]

Structure-function analysis of the EF-hand protein centrin-2 for its intracellular localization and nucleotide excision repair

Ryotaro Nishi, Wataru Sakai, Daisuke Tone, Fumio Hanaoka and Kaoru Sugasawa

SUPPLEMENTARY METHODS

Biotinylation of the XPC protein complex

Because the purified XPC protein complex tends to aggregate in solution of low ionic strength, biotinylation reactions were carried out with the protein immobilized on heparin beads. Heparin Sepharose 6 Fast Flow (GE Healthcare Biosciences) beads were washed with buffer PET [20 mM sodium phosphate (pH 7.8), 1 mM EDTA, 10% glycerol, 0.01% Triton X-100, 0.1 mM dithiothreitol (DTT), 0.25 mM phenylmethylsulfonyl fluoride (PMSF)] containing 0.3 M NaCl, and suspended in the same buffer to make 50% suspension. Fifty microliter of this suspension was added to 0.5 ml solution of the purified FLAG-AviTag-XPC/RAD23B-His complex (~50 μg/ml) in the same buffer and rotated at 4°C for at least 1 h. The beads were washed three times with 1 ml of 10 mM Tris-HCl (pH 8) and suspended in 55 μl of the same buffer. To this suspension, 10 μl each of Biomix-A (0.5 M bicine buffer, pH 8.3) and Biomix-B (100 mM ATP, 100 mM magnesium acetate, 500 μM d-biotin) solutions (both supplied with the BirA enzyme from Avidity) were added. After addition of 5 μl of the BirA biotin ligase (1 mg/ml), the mixture was incubated at 30°C for 2 h with occasional agitation. The beads were then washed 5 times with 1 ml of buffer PET containing 0.3 M NaCl and finally suspended in 100 μl of buffer PET containing 1.5 M NaCl to elute the bound protein. We confirmed that the XPC complex was recovered in a nearly quantitative manner, at least 30 to 40% of which bound to streptavidin-coated paramagnetic beads.

Preparation of the GST-centrin-2 fusion proteins

For bacterial expression of glutathione *S*-transferase (GST)-tagged centrin-2, cDNA encoding wild-type (WT) or mutant centrin-2 was cloned into the pGEX-6P-1 vector (GE Healthcare Biosciences). An *E. coli* strain BL21 (DE3) was transformed with these expression constructs and grown at 37°C in 500 ml of Super Broth medium until OD_{600} reached 0.6. Then protein expression was induced by addition of 0.5 mM isopropyl β-thiogalactopyranoside and further cultured at 30°C for 3 h. The bacterial cells were collected by centrifugation, washed twice with ice-cold 10% glycerol, and suspended in buffer D [50 mM Tris-HCl (pH 7.5), 0.5 mM EDTA, 5 mM $CaCl_2$, 2 mM DTT, 0.5 mM PMSF, 10% glycerol, a protease inhibitor cocktail] containing 1.5 M NaCl. The soluble protein fraction was obtained by sonication followed by centrifugation at 50,000 x g for 30 min. The clarified extract was loaded onto a GSTrap FF column (GE Healthcare Biosciences) equilibrated with buffer D containing 1.5 M NaCl. The column was washed extensively with the same buffer, and the bound proteins were eluted with buffer D containing 1.5 M NaCl and 10 mM glutathione. Eluted proteins were then loaded onto a HiTrap Phenyl FF column (GE Healthcare Biosciences) equilibrated with buffer D containing 1.5 M NaCl. After the column was washed with the same buffer, bound proteins were eluted with buffer D containing 0.3 M NaCl.

Immunofluorescent staining

To examine subcellular localization of centrin-2, XP4PASV cells (1 x 10^5 cells) were inoculated in a 35-mm glass bottom dish (MatTek) and cultured overnight. The cells were transfected with the expression constructs for FLAG-XPC and/or HA-centrin-2 (WT or mutant). The amounts of DNA used for transfection were as follows: 0.68 μg (FLAG-XPC), 0.34 μg (HA-centrin-2, WT), 1.36 μg (HA-centrin-2, C), and 1 μg (HA-centrin-2, M4), respectively. After additional 24-h culture, cells were washed with phosphate-buffered saline (PBS) and fixed in 5 mM EGTA/methanol at -20°C for 10 min. The following procedures were conducted at room temperature. Cells were washed twice with PBS and incubated for 30 min in 3% fetal bovine serum (FBS) in PBS to block non-specific binding of antibodies. After three times wash with PBS, cells were incubated for 1 h with primary

antibodies diluted in PBS containing 0.5% FBS and 0.05% Tween-20. Subsequently, cells were washed three times with PBS and incubated for 1 h with appropriate secondary antibodies labeled with Alexa Fluor fluorescent dyes (Life Technologies). Finally, cells were washed three times with PBS and mounted under coverslips with the Vectashield mounting medium (Vector Laboratories).

To visualize protein accumulation in local UV-damage, cells in a 35-mm glass bottom dish were irradiated with 100 J/m^2 of UVC through an isopore polycarbonate membrane filter (pore size, 5 μm: Millipore). After incubation at 37°C for 1 h, cells were washed twice with ice-cold PBS and then fixed with 1.6 % formaldehyde at 4°C for 15 min. Subsequently, cells were washed with ice-cold PBS and permeabilized with 0.5% Triton X-100 on ice for 10 min. Then the cells were treated successively with blocking solution, with primary antibodies against target proteins, and with Alexa Fluor-labeled secondary antibodies as described above. To co-visualize UV-induced cyclobutane thymine dimers (CTD), the sample was treated again with 1.6% formaldehyde at room temperature for 10 min and then with 2 M HCl at 37°C for 10 min to denature DNA. Cells were further incubated with anti-CTD antibody (TDM2) and corresponding secondary antibody, at 37°C for 30 min, respectively. Fluorescent images were observed and analyzed with an Olympus IX-71 microscope system and the MetaMorph software (Molecular Devices).

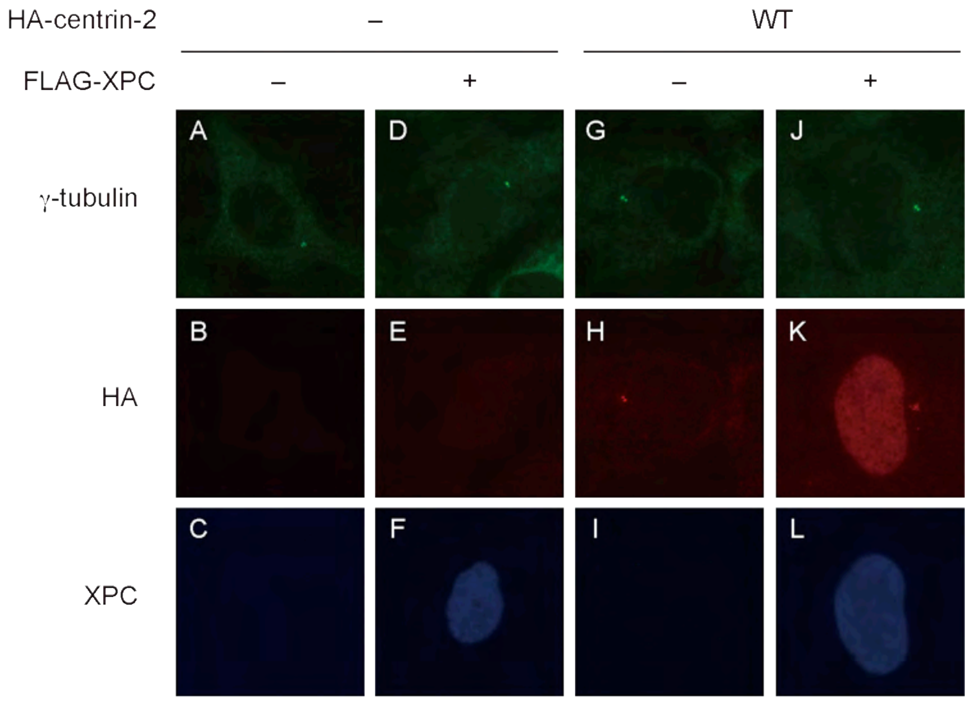

Supplementary Figure S1. Subcellular localization of the ectopically-expressed centrin-2 after global UVC irradiation. FLAG-XPC and HA-centrin-2 (WT) were transiently expressed in XP4PASV cells in various combinations. At 24 h post-transfection, cells were treated with UVC (10 J/m^2), further incubated at 37°C for 30 min, and then subjected to immunofluorescent analyses with the indicated antibodies as in Figure 2. The localization of HA-centrin-2 was essentially the same as observed in non-irradiated cells (see Figure 2, panels A-L).

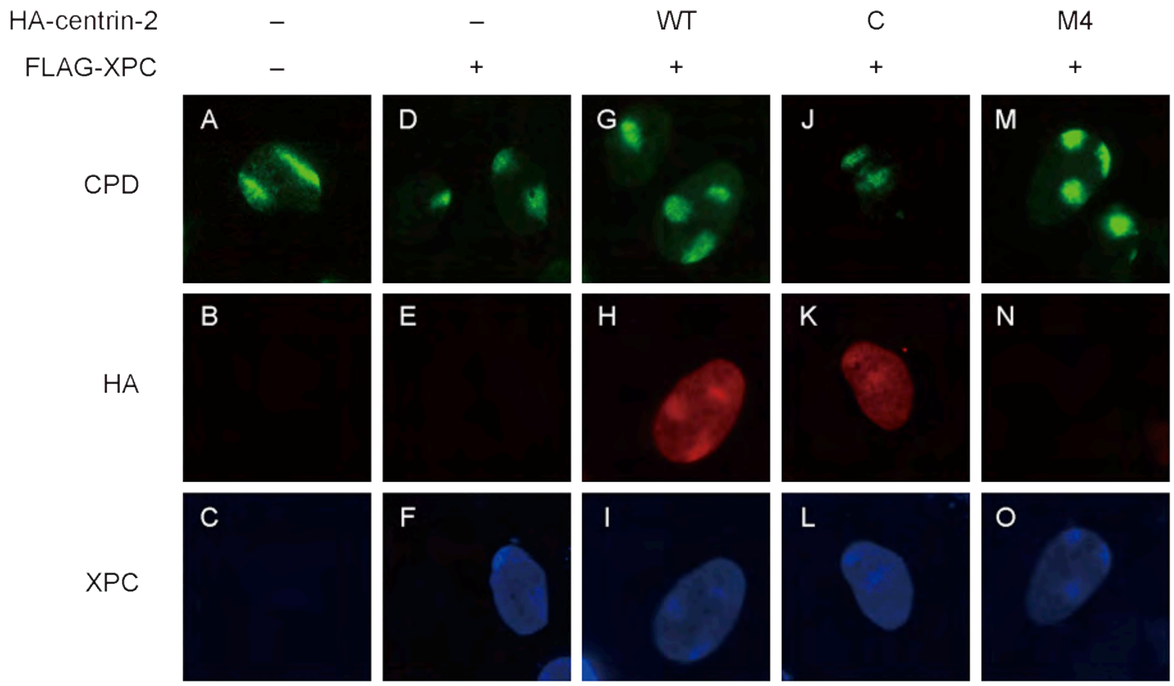

Supplementary Figure S2. The C domain, but not the N domain, of centrin-2 is responsible for its recruitment to localized UV damage. HA-centrin-2 (WT, C or M4) was transiently co-expressed with FLAG-XPC in XP4PASV cells. At 24 h post-transfection, cells were irradiated with UVC (100 J/m^2) through isopore membrane filters (pore size: 5 μm). After incubation at 37°C for 1 h, the cells were subjected to immunofluorescent staining with the indicated antibodies.

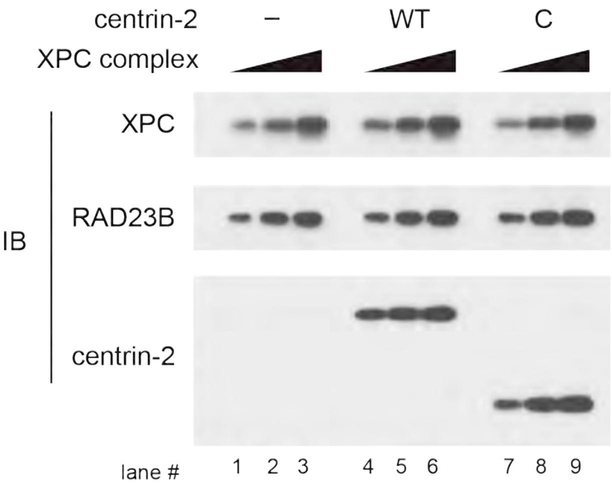

Supplementary Figure S3. Quantitative comparison of the subunits involved in the purified XPC complex used for the biochemical assays. Three different amounts of the XPC/RAD23B heterodimer (lanes 1-3) and the heterotrimer containing either WT centrin-2 (lanes 4-6) or the C domain of centrin-2 (lanes 7-9) used in Figure 4 were subjected to immunoblot analyses using the antibodies reacting with individual subunits.

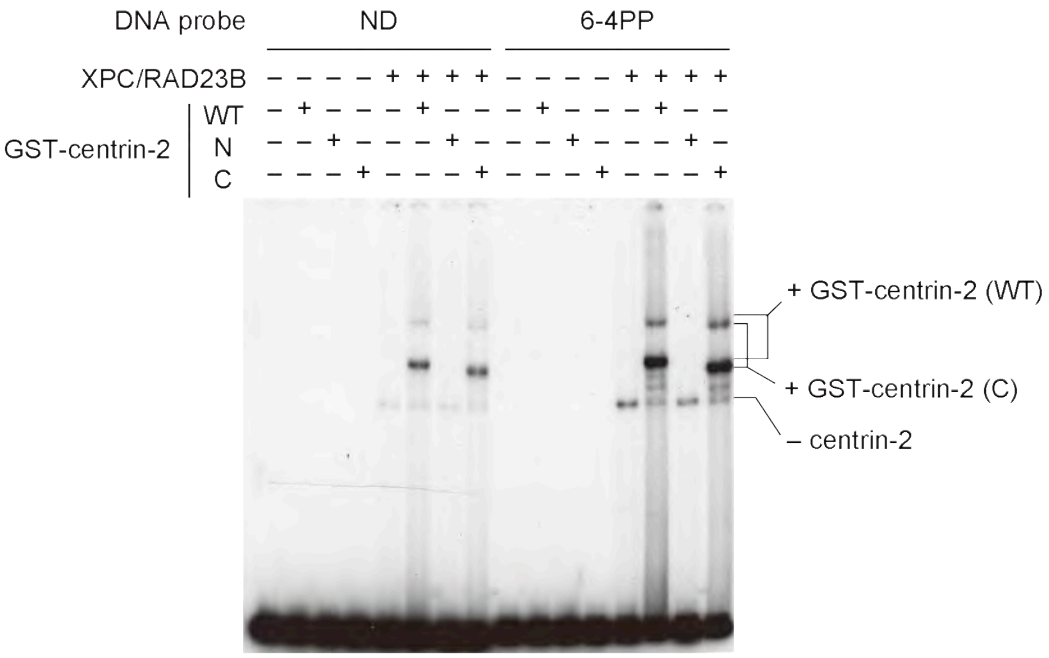

Supplementary Figure S4. Effect of GST-centrin-2 fusion proteins on damaged DNA-binding by XPC. EMSA was carried out with DNA substrates, which contained no damage (ND) or a single 6-4PP. In addition to XPC/RAD23B (4 nM), an equimolar concentration of GST-centrin-2 (WT, N or C) was separately included in the reactions as indicated. Because of the presence and possible dimerization of the GST tag, binding to WT centrin-2 or the C domain, but not N domain, caused significant mobility shifts of the DNA-XPC/RAD23B complexes unlike the results in Figure 4A.

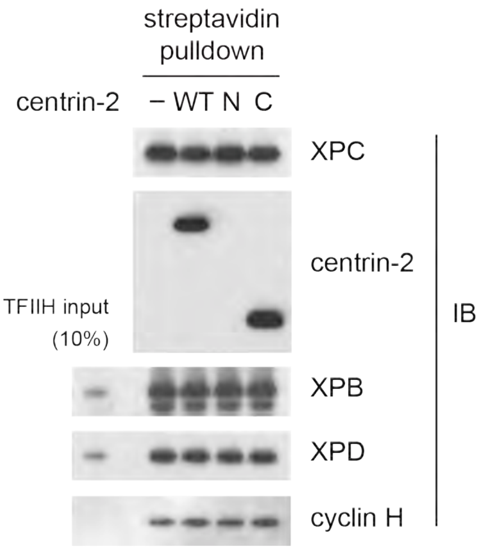

Supplementary Figure S5. TFIIH tightly interacts with XPC regardless of the presence or absence of centrin-2. Purified recombinant TFIIH was pulled down with paramagnetic beads pre-bound to XPC/RAD23B with or without centrin-2 (WT, N or C). Unlike the experiments shown in Figure 5B, binding of TFIIH and the following washes were carried out with buffer containing 0.3 M NaCl, instead of 0.15 M. The co-precipitated proteins were detected with the indicated antibodies.

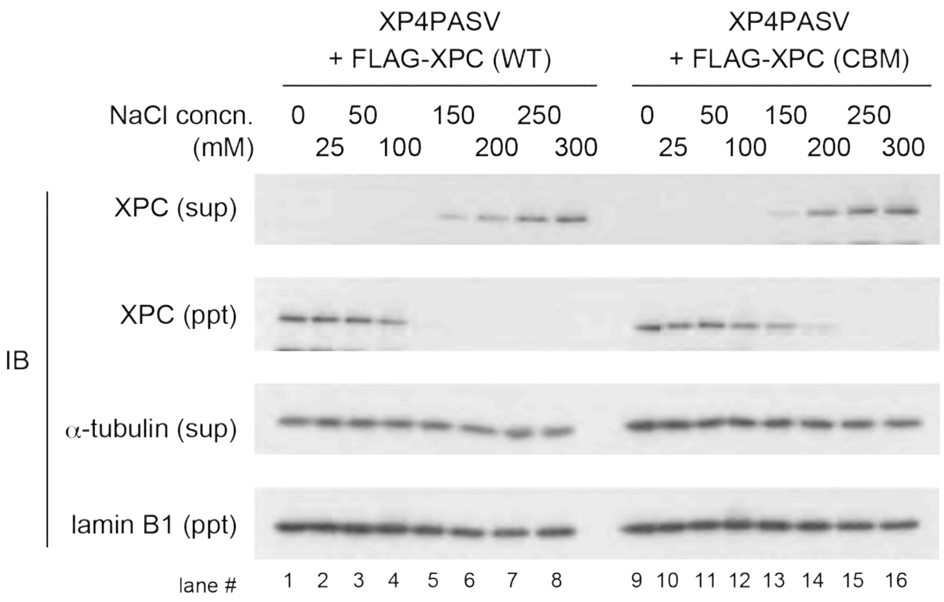

Supplementary Figure S6. Salt extraction profiles of the wild-type XPC and XPC (CBM) proteins. The transformed cell lines of XP4PASV used for the ChIP experiments (Figure 6), which stably expressed FLAG-XPC (wild-type or CBM) at physiological levels, were extracted with CSK buffer containing various concentrations of NaCl as indicated. After soluble fractions (sup) were recovered by centrifugation, the remaining insoluble fractions (ppt) were suspended by sonication in the same buffer. The resulting fractions were subjected to immunoblot analyses using the indicated antibodies.
